# Dietary fermented soybean meal replacement alleviates diarrhea in weaned piglets challenged with enterotoxigenic *Escherichia coli* K88 by modulating inflammatory cytokine levels and cecal microbiota composition

**DOI:** 10.1186/s12917-020-02466-5

**Published:** 2020-07-14

**Authors:** Wenwen Wang, Yuan Wang, Xiran Hao, Yuanxiao Duan, Ziqi Meng, Xiaoping An, Jingwei Qi

**Affiliations:** 1 College of Animal Science, Inner Mongolia Agricultural University, 010018 Hohhot, China; 2 Inner Mongolia Herbivorous Livestock Feed Engineering and Technology Research Center, 010018 Hohhot, China

**Keywords:** Fermented soybean meal, Inflammatory cytokines, Cecal microbiota, Weaned piglets, Enterotoxigenic *Escherichia coli* K88

## Abstract

**Background:**

Impaired gut microbiota leads to pathogenic bacteria infection, pro-inflammatory response and post-weaning diarrhea. Enterotoxigenic *Escherichia coli* (ETEC) K88 is a major cause of post-weaning diarrhea in weaned piglets. Fermented soybean meal (FSBM) could relieve diarrhea, alleviate inflammatory response, and modulate gut microbiota of weaned piglets. We used ETEC K88-challenged weaned piglet model to investigate the effects of FSBM on the growth performance, inflammatory response and cecal microbiota. Twenty-four crossbred piglets (6.8 ± 0.5 kg; 21 ± 2 days of age) were allotted into 2 treatment fed the diets with or without FSBM (6% at the expense of soybean meal). Six weaned piglets in each diet treatment were challenged by ETEC K88 (1 × 10^9^ CFU/piglets) on day 15. The experimental period lasted for 20 days.

**Results:**

The ETEC K88 challenge decreased (*p* < 0.05) fecal consistency and plasma interleukin-10 (IL-10) concentration, while increased (*p* < 0.05) average daily feed intake (ADFI) and plasma tumor necrosis factor-α (TNF-α), interleukin-1β (IL-1β) and interleukin 6 (IL-6) concentrations. After ETEC K88 challenge, dietary FSBM replacement increased (*p <* 0.05) final body weight (BW), average daily gain (ADG), ADFI, and fecal consistency, but decreased feed conversion ratio (FCR). The plasma IL-10 concentration of weaned piglets fed FSBM was higher (*p <* 0.05), while IL-1β, IL-6 and TNF-α concentrations were lower (*p <* 0.05). Dietary FSBM replacement attenuated the increase of plasma TNF-α concentration and the decrease of ADG induced by ETEC K88 challenge (*p <* 0.05). High-throughput sequencing of 16S rRNA gene V4 region of cecal microbiota revealed that ETEC K88 challenge increased (*p <* 0.05) *Campylobacter* relative abundance on genus level. Dietary FSBM replacement resulted in higher (*p <* 0.05) relative abundances of Bacteroidetes and *Prevotellaceae_NK3B31_group*, and lower (*p <* 0.05) relative abundances of Proteobacteria and *Actinobacillus*. Furthermore, dietary FSBM replacement relieved the increase of *Escherichia-Shigella* relative abundance in weaned piglets challenged by ETEC K88 (*p <* 0.05).

**Conclusions:**

Dietary FSBM replacement improved growth performance and alleviated the diarrhea of weaned piglets challenged with ETEC K88, which could be due to modulation of cecal microbiota composition and down-regulation of inflammatory cytokines production.

## Background

The gastrointestinal tract (GIT) of animal harbors vast and complex microorganisms, including bacteria, fungi, protozoa, and archaea [1]. The gut microbiota plays important roles in preventing colonization of pathogenic bacteria, modulating immune response and maintaining GIT homeostasis [1]. The structure and composition of gut microbiota is impacted by several factors, such as genotype, age, diet, environment, and stress [2]. In swine production, early weaning technology is widely used to meet production goals [3]. The weaning stress disrupts the gut microbiota of piglets, leading to pathogenic bacteria infection, pro-inflammatory response and post-weaning diarrhea [4]. The Enterotoxigenic *Escherichia coli* (ETEC) K88 is considered to be major pathogen of post-weaning diarrhea in weaned piglets [5]. Traditionally, in-feed antibiotics are used to control post-weaning diarrhea and improve growth performance of weaned piglets. However, it is suggested that the long-term use of antibiotics altered structure and composition of gut microbiota and down-regulated the expression of genes related to immune response [5, 6]. As antibiotics alternatives, prebiotics, probiotics, synbiotics, and fermented feed have been demonstrated to modulate the gut microbiota and improve piglet’s health [2].

Fermented soybean meal (FSBM) is manufactured from soybean meal (SBM) by fungal and bacterial strains [7]. The organic acids, antibacterial peptides, oligosaccharides and other “minor” components, having beneficial effects on piglet’s health, are produced by fermentation [7]. Xie et al. (2017) found that FSBM improved average daily gain (ADG), average daily feed intake (ADFI) and feed conversion ratio (FCR) and reduced diarrhea incidence of piglets by increasing gut bacterial diversity and relative abundance of butyrate-producing bacteria in the cecum [8]. Zhang et al. (2018) observed that FSBM changed microbiota composition in large intestine and improved ADG and FCR of piglets [9]. In addition, it has been reported that the mRNA expression of pro-inflammatory cytokines (interleukin-4 (IL-4) and interleukin 6 (IL-6)) in jejunum and ileum of piglets was reduced by feeding FSBM [10]. However, the influence of FSBM on growth performance, diarrhea incidence and gut microbiota composition in ETEC K88 challenged weaned piglets is poorly investigated. Kiers et al. (2006) reported that feeding ETEC-infected piglets with fermented soybeans resulted in enhancement in intestinal water and electrolyte absorption [11].

In the present study, it was hypothesized that dietary FSBM replacement could improve growth performance and alleviate diarrhea by modulating cecal microbiota and reducing inflammatory response in piglets challenged with ETEC K88. To such purpose, the effects of FSBM on growth performance, fecal consistency, plasma IL-6, interleukin-1β (IL-1β), interleukin-10 (IL-10), and tumor necrosis factor α (TNF-α) concentrations and cecal microbiota composition were investigated in ETEC K88-challenged weaned piglets.

## Results

### Chemical composition of SBM and FSBM

The chemical composition of SBM and FSBM used in this study is shown in Table 1. The pH value and β-conglycinin and glycinin concentrations in FSBM were lower than in SBM. The fermentation process changed peptide size distribution of SBM. The percentage of large peptides (60 kDa and higher) and middle peptides (20 to 60 kDa) were lower in FSBM than in SBM, while the percentage of small peptides (20 kDa and lower) was higher.

**Table 1:** Chemical composition of soybean meal (SBM) and fermented soybean meal (FSBM) (as-fed basis)^1^

	SBM	FSBM
pH	6.5	4.9
β-conglycinin (mg/g)	72.8	42.3
Glycinin (mg/g)	89.7	38.7
Peptide size distribution (%)		
60 kDa and higher	25.3	8.9
20 to 60 kDa	49.4	45.1
20 kDa and lower	25.3	46.0

### Growth performance and fecal consistency

The effects of experimental treatments on growth performance and fecal consistency of weaned piglets are summarized in Table 2. During the first 14 days of trial, there was no difference in the final body weight (BW), ADG, ADFI, and FCR of weaned piglets between FSBM and SBM group. After ETEC K88 challenge, weaned piglets given FSBM had higher (*p* < 0.05) final BW, ADFI and ADG, while lower (*p* < 0.05) FCR. The ETEC K88 challenge had no effect on the growth performance of weaned piglets. There was an interaction between dietary FSBM replacement and ETEC K88 challenge for ADG of weaned piglets (*p <* 0.05). The weaned piglets given FSBM without ETEC K88 challenge had a greater ADG. The fecal consistency of weaned piglets was decreased (*p* < 0.01) by ETEC K88 challenge, while increased (*p* < 0.01) by dietary FSBM replacement.

**Table 2:** Effects of dietary fermented soybean meal (FSBM) replacement and/or Enterotoxigenic *Escherichia coli* (ETEC) K88 infusion on growth performance and fecal consistency in weaned piglets ^1, 2^

Item	*-* ETEC K88		+ ETEC K88		SEM	*P*-value		
	- FSBM	+ FSBM	- FSBM	+ FSBM		FSBM	ETEC K88	FSBM × ETEC K88
Before challenge (d 1–14)								
Initial BW (kg)	6.8	7.0	-	-	0.10	0.32		
Final BW (kg)	11.3	11.8	-	-	0.19	0.21		
ADFI (g)	466.2	487.4	-	-	9.69	0.29		
ADG (g)	321.1	341.8	-	-	11.02	0.36		
FCR	1.47	1.44	-	-	0.036	0.71		
After challenge (d 15–20)								
Initial BW (kg)	11.0	12.0	11.6	11.5	0.19	0.24	0.85	0.15
Final BW (kg)	12.5	14.4	13.5	13.6	0.26	0.04	0.87	0.07
ADFI (g)	709.4	861.9	855.5	892.7	23.57	0.03	0.04	0.16
ADG (g)	259.0^c^	408.8^a^	313.0^bc^	355.3^ab^	16.44	< 0.01	0.99	0.04
FCR	2.88	2.12	2.77	2.52	0.117	0.03	0.51	0.24
Fecal consistency^3^	2.90	3.05	2.13	2.61	0.086	< 0.01	< 0.01	0.13

^1^*n* = 12 before challenge; *n* = 06 after challenge

^2^ - ETEC K88 = infusing the phosphate buffer saline; + ETEC K88 = infusing the ETEC K88; - FSBM = control diet with 24% soybean meal; + FSBM = test diet with 6% FSBM added at the expense of soybean meal

^3^ Faeces were scored as follows: 1, watery, mucous-like; 2, loose, semi-liquid; 3, soft, partially formed; 4, slightly soft; and 5, hard. The fecal consistency was calculated as [(Σ fecal scores for 6 days ETEC K88 challenge)/n]

^a,b,c^ Mean values within a row with unlike superscript letters were significantly different (*p <* 0.05)

*BW* Body weight; *ADFI* Average daily feed intake; *ADG* Average daily gain; *FCR* Feed conversion ratio

### Plasma cytokines concentrations

As shown in Table 3, the weaned piglets fed with FSBM had lower (*p <* 0.05) plasma TNF-α, IL-1β, and IL-6 concentrations, and higher (*p* < 0.01) plasma IL-10 concentration compared with SBM. The ETEC K88 challenge increased (*p <* 0.05) the plasma TNF-α, IL-1β, and IL-6 concentrations, and decreased (*p* < 0.01) the plasma IL-10 concentration. There was an FSBM × ETEC K88 interaction for plasma TNF-α concentration (*p <* 0.05). In the weaned piglets challenged by ETEC K88, dietary FSBM replacement could alleviate the effect of ETEC K88 challenge on plasma TNF-α concentration.

**Table 3:** Effects of dietary fermented soybean meal (FSBM) replacement and/or Enterotoxigenic *Escherichia coli* (ETEC) K88 infusion on plasma cytokines concentrations in weaned piglets ^1,2^

Item	*-* ETEC K88		+ ETEC K88		SEM	*P*-value		
	- FSBM	+ FSBM	- FSBM	+ FSBM		FSBM	ETEC K88	FSBM × ETEC K88
TNF-α (ng/L)	146.1^c^	133.0^c^	234.4^a^	179.7^b^	9.73	< 0.01	< 0.01	0.03
IL-1β (ng/L)	15.1	13.2	18.9	15.6	0.67	0.03	0.01	0.52
IL-6 (ng/L)	13.1	9.6	15.1	13.8	0.30	0.02	< 0.01	0.41
IL-10 (ng/L)	96.3	112.0	80.4	95.9	3.31	< 0.01	< 0.01	0.97

^1^*n* = 06

^2^ - ETEC K88 = infusing the phosphate buffer saline; + ETEC K88 = infusing the ETEC K88; - FSBM = control diet with 24% soybean meal; + FSBM = test diet with 6% FSBM added at the expense of soybean meal

^a,b,c^ Mean values within a row with unlike superscript letters were significantly different (*p <* 0.05)

*TNF-α* Tumor necrosis factor-α; *IL-1β* Interleukin-1β; *IL-6* Interleukin-6; *IL-10* Interleukin-10

### Sequence depth and bacterial diversity of cecal microbiota

We sequenced the V4 region of the 16S rRNA gene to identify the cecal microbiota composition in weaned piglets. Across all 24 samples, a total of 2087447 raw tags and an average of 86977 ± 7459 raw tags per sample were generated. After size filtering, quality control and chimera removal, a total of 1975170 effective tags were obtained, and each cecal digesta sample produced 82299 ± 7126 effective tags. Based on a sequence similarity of greater than 97%, 35448 operational taxonomic units (OTUs) were defined and classified into 2 kingdoms, 48 phyla, 109 class, 170 orders, 315 families and 639 genera.

The alpha diversity indices of cecal microbiota in weaned piglets are shown in Table 4. Dietary FSBM replacement increased (*p <* 0.05) the Observed_species, Shannon, Chao1, and ACE indexes. The ETEC K88 challenge led to a decrease (*p <* 0.05) in Observed_species, Chao1, and ACE indexes, but did not affect the Shannon index. There were interactions between dietary FSBM replacement and ETEC K88 challenge for the Observed_species, Chao1, and ACE indexes (*p* < 0.01). The piglets given FSBM without ETEC K88 challenge had higher Observed_species, Chao1 and ACE indexes.

**Table 4:** Differences in the alpha diversity of the cecal microbiota of weaned piglets ^1,2^

Item	*-* ETEC K88		+ ETEC K88		SEM	*P*-value		
	- FSBM	+ FSBM	- FSBM	+ FSBM		FSBM	ETEC K88	FSBM × ETEC K88
Observed_species	1102.33^b^	2131.67^a^	1041.17^b^	854.00^b^	130.927	0.02	< 0.01	< 0.01
Shannon	6.68	7.69	6.63	6.78	0.141	0.02	0.05	0.08
Simpson	0.97	0.98	0.96	0.97	0.003	0.07	0.14	0.49
Chao1	1237.73^b^	2424.03^a^	1174.07^b^	940.28^b^	151.793	0.03	< 0.01	< 0.01
ACE	1266.63^b^	2506.32^a^	1206.67^b^	951.99^b^	158.367	0.03	< 0.01	< 0.01

^1^*n* = 06

^2^ - ETEC K88 = infusing the phosphate buffer saline; + ETEC K88 = infusing the ETEC K88; - FSBM = control diet with 24% soybean meal; + FSBM = test diet with 6% FSBM added at the expense of soybean meal

^a,b^ Mean values within a row with unlike superscript letters were significantly different (*p <* 0.05)

**Fig. 1:**
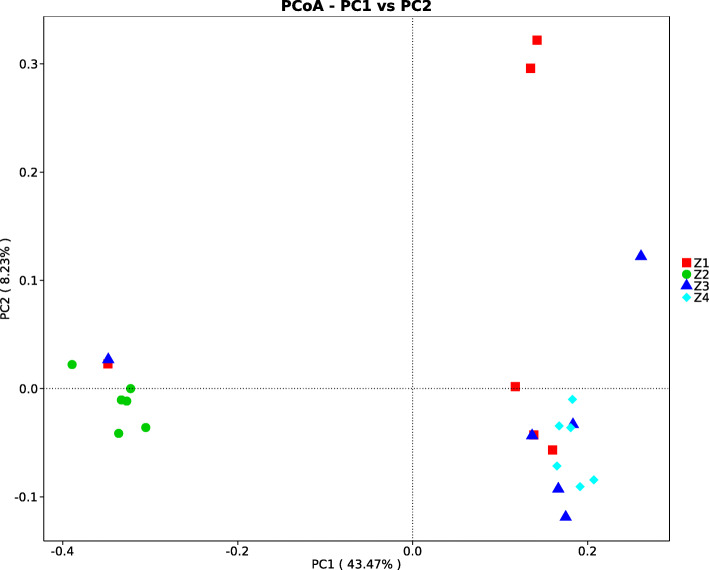
Principal coordinate analysis (PCoA) of the bacterial composition similarity based on the unweighted UniFrac distances in the cecal digesta of four groups. Z1: SBM-PBS group; Z2: FSBM-PBS group; Z3: SBM-ETEC K88 group; Z4: FSBM-ETEC K88 group

For beta diversity analysis, PCoA based on Unweighted UniFrac distances was performed in cecal microbiota collected from weaned piglets. The results (Fig. 1) revealed that all samples fell into two clusters. The microbiota from weaned piglets of FSBM-phosphate-buffered saline (PBS) group was separated from the other three groups.

### Bacterial composition and relative abundance of cecal microbiota

The relative abundances of the 10 most abundant phyla are presented in Table 5. Firmicutes, Bacteroidetes, and Proteobacteria were the three predominant phyla and accounted for more than 89% of cecal microbiota in weaned piglets. The consumption of FSBM increased (*p <* 0.05) the relative abundances of Bacteroidetes, Acidobacteria, and Chloroflexi, and decreased (*p <* 0.05) the relative abundance of Proteobacteria. The ETEC K88 challenge decreased (*p <* 0.05) the relative abundance of Tenericutes, Acidobacteria, and Chloroflexi. There were interactions between dietary FSBM replacement and ETEC K88 challenge for the relative abundances of Verrucomicrobia, Acidobacteria, and Chloroflexi (*p <* 0.05). The relative abundances of Verrucomicrobia, Acidobacteria, and Chloroflexi were increased in piglets receiving FSBM without the ETEC K88 challenge.

**Table 5:** Relative abundance of cecal microbiota at the phylum level of weaned piglets^1,2^

Item	*-* ETEC K88		+ ETEC K88		SEM	*P*-value		
	- FSBM	+ FSBM	- FSBM	+ FSBM		FSBM	ETEC K88	FSBM × ETEC K88
Firmicutes	51.107	43.479	45.980	46.081	1.5906	0.250	0.696	0.238
Bacteroidetes	27.838	35.241	28.770	39.087	1.6622	0.006	0.419	0.620
Proteobacteria	15.367	10.719	20.454	12.415	1.4896	0.031	0.229	0.542
Spirochaetes	1.023	2.691	1.366	0.583	0.3400	0.496	0.182	0.069
Cyanobacteria	2.461	1.937	1.367	0.858	0.3543	0.476	0.142	0.991
Verrucomicrobia	0.115^b^	1.272^a^	0.446^ab^	0.097^b^	0.1848	0.241	0.221	0.036
Tenericutes	1.026	1.231	0.553	0.516	0.1224	0.712	0.015	0.595
Acidobacteria	0.137^b^	0.770^a^	0.127^b^	0.020^b^	0.0741	0.007	< 0.001	< 0.001
Chloroflexi	0.130^b^	0.719^a^	0.120^b^	0.017^b^	0.0680	0.005	< 0.001	< 0.001
Fusobacteria	0.006	0.003	0.143	0.005	0.0342	0.318	0.322	0.336

^1^*n* = 06

^2^ - ETEC K88 = infusing the phosphate buffer saline; + ETEC K88 = infusing the ETEC K88; - FSBM = control diet with 24% soybean meal; + FSBM = test diet with 6% FSBM added at the expense of soybean meal

^a,b^ Mean values within a row with unlike superscript letters were significantly different (*p <* 0.05)

The relative abundances of the 10 most abundant genera are presented in Table 6. The relative abundance of *Prevotellaceae_NK3B31_group* in the cecal digesta was increased (*p <* 0.05) by dietary FSBM replacement, whereas the relative abundance of *Actinobacillus* was decreased (*p <* 0.05). The ETEC K88 challenge increased *Campylobacter* relative abundance (*p <* 0.05). Furthermore, there was an interaction between dietary FSBM replacement and ETEC K88 challenge with respect to *Escherichia-Shigella* relative abundance (*p <* 0.05). In the weaned piglets challenged by ETEC K88, dietary FSBM replacement could relieve the effect of ETEC K88 challenge on *Escherichia-Shigella* relative abundance.

**Table 6:** Relative abundance of cecal microbiota at the genus level of weaned piglets ^1,2,3^

Item	*-* ETEC K88		+ ETEC K88		SEM	*P*-value		
	- FSBM	+ FSBM	- FSBM	+ FSBM		FSBM	ETEC K88	FSBM × ETEC K88
*Alloprevotella*	6.707	4.767	8.110	10.445	0.9549	0.915	0.067	0.256
*Escherichia-Shigella*	3.410^b^	4.427^ab^	8.517^a^	2.022^b^	0.9126	0.109	0.417	0.032
*Prevotellaceae_NK3B31_group*	1.930	8.043	2.960	3.155	0.8516	0.047	0.210	0.061
*Campylobacter*	1.675	0.317	6.153	3.718	0.8505	0.231	0.018	0.730
*Actinobacillus*	6.082	1.425	3.118	1.463	0.6728	0.014	0.225	0.213
*Faecalibacterium*	5.537	2.135	3.442	4.647	0.6792	0.422	0.878	0.101
*Prevotella_9*	3.774	2.834	3.739	5.008	0.5646	0.889	0.368	0.352
*Ruminococcaceae_UCG-005*	2.954	2.939	1.511	2.005	0.4301	0.788	0.192	0.775
*Clostridium_sensu_stricto_1*	5.360	2.550	3.108	2.001	0.5356	0.063	0.175	0.403
*Anaerovibrio*	2.232	2.819	3.116	3.113	0.3486	0.693	0.430	0.690

^1^*n* = 06

^2^ - ETEC K88 = infusing the phosphate buffer saline; + ETEC K88 = infusing the ETEC K88; - FSBM = control diet with 24% soybean meal; + FSBM = test diet with 6% FSBM added at the expense of soybean meal

^a,b^ Mean values within a row with unlike superscript letters were significantly different (*p <* 0.05)

## Discussion

Post-weaning diarrhea in weaned piglets is one of the most serious threats for the swine production. It is associated with the colonization of ETEC in the piglet intestine, and characterized by gut dysbiosis, inflammation, growth retardation, and sudden death [12, 13]. Inclusion of FSBM in feed has been demonstrated to reduce diarrhea incidence, alleviate inflammation, and modulate structure and composition of gut microbiota in piglets [8–10]. Therefore, we used ETEC K88-challenged weaned piglet model to study the effects of FSBM on protecting piglets against diarrhea by modulating gut microbiota and alleviating pro-inflammatory response.

In the present study, FSBM had lower pH value and lower glycinin and β-conglycinin contents, while higher small-size peptides content. Three probiotic strains (*S. thermophilus, S. cerevisiae* and *B. subtilis* MA 139) were used for fermentation of SBM. *S. cerevisiae* created an anaerobic environment for the growth of anaerobic bacteria (*S. thermophilus* and *B. subtilis* MA 139). Subsequent to the production of organic acids by anaerobic bacteria, the pH value of FSBM was reduced [14]. In addition, these three probiotic strains produced a variety of proteases during the fermentation [14]. Therefore, soybean proteins (β-conglycinin and glycinin) were degraded into small-size peptides and amino acids, which were easily absorbed by weaned piglets. Similar to our findings, it has also been reported that fermentation of SBM with *B. subtilis*, *S. cerevisiae* and *A. niger* increased the amount of lactic acid, decreased pH value, and degraded anti-nutritional factors [15].

In the present study, the fecal consistency was decreased in the ETEC K88-challenged weaned piglets, confirming that the model was successful. Dietary FSBM replacement increased the growth performance and fecal consistency of weaned piglets post-challenge. These results are consistent with Kiers et al. [11] and Xie et al. [8], who reported that FSBM reduced post-weaning diarrhea and increased ADG of weaned piglets. We speculated that organic acids produced by anaerobic bacteria during the fermentation of SBM may reduce the intestine pH of weaned piglets, suppress pathogenic bacteria proliferation and consequently alleviate diarrhea [16, 17]. It is confirmed by reduced the relative abundance of *Escherichia-Shigella* and *Clostridium_sensu_stricto_1* of weaned piglets fed with FSBM in this study. Surprisingly, there was a big drop (321.1 to 259.0 g) in ADG of weaned piglets fed with SBM before and after ETEC K88 challenge, which need further study.

After ETEC K88 challenge, the bacterium colonizes in the intestine of weaned piglets and produces enterotoxin, which leading to inflammation by stimulating synthesis of pro-inflammatory cytokines, such as IL-6, IL-1β, and TNF-α [18, 19]. This is in agreement with our finding that plasma TNF-α, IL-1β, and IL-6 levels increased and IL-10 level decreased in weaned piglets challenged by ETEC K88. Dietary FSBM replacement decreased TNF-α, IL-1β, and IL-6 concentrations and increased IL-10 concentration in the plasma of weaned piglets. Moreover, under ETEC K88-challenged condition, FSBM alleviated the increase of plasma TNF-α concentration of weaned piglets. This finding is consistent with Zhang et al. [10], who reported that piglets fed a diet containing 10% FSBM had reduced IL-4 and IL-6 transcripts and increased IL-10 transcript in ileum. The FSBM contained more probiotics and organic acids compared with SBM. It has been reported that probiotics and organic acids could regulate the production of cytokines and relieve ETEC K88-induced inflammation by suppressing the ETEC K88 proliferation and decreasing enterotoxin release [20–23]. This finding was also confirmed by the results of high-throughput sequencing, which shown that FSBM decreased the population of inflammation-related bacteria.

Researches have shown that diarrhea and inflammation are associated with altered gut microbiota [24, 25]. In this study, we used high-throughput sequencing of the 16S rRNA gene V4 region to investigate the effects of dietary FSBM replacement and ETEC K88 challenge on structure and composition of cecal microbiota. We observed that ETEC K88 challenge decreased the numbers of total bacteria (Observed_species), richness estimators (Chao1 and ACE) and diversity estimator (Shannon), while did not affect the diversity estimator (Simpson) of cecal microbiota. It is suggested that loss of diversity dramatically increased the risk of gastrointestinal diseases, such as inflammatory bowel disease and functional dyspepsia [26, 27]. As we expected, administration of FSBM significantly enhanced cecal microbiota richness and diversity of weaned piglets. These results are in line with a previous study that has reported increased both the richness and diversity of cecal and colonic microbiota in weaned piglets fed with fresh FSBM [8]. Interestingly, in the weaned piglets challenged by ETEC K88, dietary FSBM replacement did not alleviate the decrease in cecal microbiota richness and diversity. It is possible that the relative abundances of *Campylobacter* and *Escherichia-Shigella* were reduced by FSBM, thus the reduced relative abundances of these pathogenic bacteria species may have resulted in the lack of effects on cecal microbiota richness and diversity.

In this study, the ETEC K88 challenge reduced the relative abundances of Tenericutes, Acidobacteria and Chloroflexi in cecum digesta of weaned piglets. It was reported that Acidobacteria was less abundant in the gut microbiota of primary biliary cirrhosis patients compared with healthy controls [28]. Dietary FSBM replacement significantly improved the relative abundances of Bacteroidetes, Acidobacteria, and Chloroflexi, and decreased the relative abundance of Proteobacteria. A similar result was also observed by Xie et al. [8], that the replacement of SBM by fresh FSBM increased Firmicutes relative abundance and decreased Bacteroidetes and Proteobacteria relative abundances. The Proteobacteria, including *Escherichia coli*, *Salmonella*, *Campylobacter*, and *Helicobacter*, was strongly associated with intestinal inflammation [29]. Moreover, dietary FSBM replacement relieved the increase of Verrucomicrobia relative abundance induced by ETEC K88 challenge in this study. Previous research provided some evidence that Verrucomicrobia appeared to contribute to inflammation as their abundance bloomed in DSS-induced colitis mice [30]. Collectively, these findings illustrated that the anti-inflammation effects of FSBM could be related to modulation of Proteobacteria, Acidobacteria and Verrucomicrobia relative abundances in the cecum.

The ETEC K88 challenge enhanced the relative abundances of *Alloprevotella* and *Campylobacter* in this study. *Campylobacter* had been observed in greater numbers in patients with inflammatory bowel disease compared with healthy controls [31, 32]. Dietary FSBM replacement increased the relative abundance of *Prevotellaceae_NK3B31_group* and decreased the relative abundances of *Actinobacillus* and *Clostridium_sensu_stricto_1* in the cecum of weaned piglets. Moreover, dietary FSBM replacement alleviated the increase of *Escherichia-Shigella* relative abundance and the decrease of *Prevotellaceae_NK3B31_group* abundance induced by ETEC K88 challenge. As reported by Dou et al. [4], the higher relative abundance of *Prevotellaceae_NK3B31_group*, which belonged to Prevotellaceae, provided a benefit to healthy pigs. Moreover, Li et al. [23] reported that dietary organic acids supplementation increased *Prevotellaceae_NK3B31_group* abundances and reduced diarrhea incidence. *Actinobacillus pleuropneumoniae* was reported to cause respiratory disease in finishing pigs [33]. Some members of *Clostridium* and *Escherichia-Shigella* were clearly related to necrotizing enterocolitis [34]. Thus, FSBM could reduce diarrhea incidence and relieve inflammation in the weaned piglets challenged by ETEC K88, which might be due to lower proportion of *Escherichia-Shigella*, *Clostridium_sensu_stricto_1*, *Campylobacter* and *Actinobacillus* in the cecal digesta. Inconsistent with current study, Xie et al. [8] and Zhang et al. [9] suggested that replacement of SBM by fresh FSBM reduced the relative abundances of *Escherichia*, *Streptococcus* and *Stenotrophomonas* and increased the relative abundances of *Prevotella*, *Roseburia*, *Feacalibacterium* and *Lactobacillus* in cecum of weaned piglets. The difference between our findings and previous reports might reflect that the microbiota profiles response to FSBM supplementation were starter strains, pH and antigens content as well as animal physiology dependent.

## Conclusions

In summary, ETEC K88 challenge led to higher fecal consistency, pro-inflammatory cytokines (TNF-α, IL-1β, and IL-6) production and *Campylobacter* relative abundance. The final BW, ADG, ADFI, and FCR of weaned piglets were improved by FSBM. Dietary FSBM replacement decreased TNF-α, IL-1β, and IL-6 concentrations and increased IL-10 concentration in the plasma of weaned piglets. In addition, dietary FSBM replacement increased relative abundances of Bacteroidetes and *Prevotellaceae_NK3B31_group* and decreased relative abundances of Proteobacteria and *Actinobacillus*. These findings suggest that dietary FSBM replacement improved growth performance and alleviated diarrhea of weaned piglets, which could be due to modulation of cecal microbiota composition and down-regulation of inflammatory cytokines production.

## Methods

### Animal care

All experimental protocols for this study were approved by the Inner Mongolia Agricultural University Animal Care and Use Committee. The animal euthanasia, sample collection and carcass disposal procedure were in strict accordance with the requirement of the Ethics Procedures and Guidelines of the People’s Republic of China.

### Preparation of bacterial strains

*Streptococcus thermophilus* (CGMCC No. 1.2471) was cultured in de Man, Rogosa and Sharp media at 37 °C for 24 h. *Saccharomyces cerevisae* (CGMCC No. 2.1793) and *Bacillus subtilis* MA139 were grown in yeast peptone dextrose and mixed nutrition broth in a rotary shaker (225 rpm) at 30 °C for 24 h, respectively. After incubation, bacterial cells were washed twice with sterile saline solution and inoculated to have a final inoculant of 1 × 10^7^ CFU/mL (i.e. the concentration of each strain was 1 × 107 CFU/mL). Equal volumes of *S. thermophilus*, *S. cerevisiae*, and *B. subtilis* MA139 were mixed as liquid starter culture for SBM fermentation. The ETEC K88 strain (serotype O149:K88; Laboratory of Animal Nutrition and Health and Key Laboratory of Agro-Ecology) was grown in Luria-Bertani broth for 24 h at 37℃ with shaking. Following incubation, the bacterial cells were harvested by centrifugation at 3000 × *g* for 10 min at 4℃, washed by PBS, and resuspended in PBS (10^9^ CFU/mL). The ETEC K88 solution was stored at 4℃ for further use.

### Fermentation of soybean meal

The SBM was obtained from Yihai Kerry Investment Co., Ltd. (Shanghai, China). Fermentation of SBM was conducted following the method described by Wang et al. [35]. Briefly, SBM was milled to pass through a 1.0-mm screen. The neutral protease and acid protease (Finnico; Bosar Biotechnology, Beijing, China) used in this study are commercial enzymes produced by submerged fermentation of *Bacillus subtilis* and *Aspergillus niger*, respectively. Neutral protease and acid protease (50,000 IU/g) were mixed in 3:1 ratio to prepare the protease mixture. The SBM was mixed with liquid starter culture (10% v/w), 0.5% (w/w) brown sugar and 0.3% (w/w) protease mixture. Then sterile distilled water was added to achieve initial moisture content (40%, w/w). Fermentation of SBM was conducted at 40℃ for 5 days in Multi-layer polythene bags equipped with a gas-pressure opening valve. After fermentation, FSBM was dried with hot air, grounded with a hammer mill (1.0 mm) and frozen until mixed in the diets.

To determine pH, 10 g of SBM and FSBM were dissolved in 100 mL distilled water. After centrifuging at 4000 × ***g*** for 5 min, the pH of the supernatant was measured. The contents of glycinin and β-conglycinin in SBM and FSBM were analyzed with commercially available enzyme-linked immunosorbent assay (ELISA) kits in accordance with the manufacturer’s protocol (Beijing Longkefangzhou Bio-Engineering Technology Co., Ltd, Beijing, China). Peptide size distribution of SBM and FSBM was performed by Tricine-SDS-PAGE according to the method of Wang et al. [35].

### Animals, experimental design and diets

A total of 24 crossbred piglets (Duroc × Landrace × Large White, 6.8 ± 0.5 kg, 21 ± 2 days of age) were provided by Hailiutu experimental farm of Inner Mongolia Agricultural University (Hohhot, Inner Mongolia, China). The piglets were blocked by sex and allocated into 1 of 2 groups, including a - FSBM group (control diet with 24% SBM, *n* = 12) and a + FSBM group (test diet with 6% FSBM added at the expense of SBM, *n* = 12). The piglets were fed with the experimental diets for 14 day before ETEC K88 challenge. Before 12 piglets were challenged with ETEC K88, the experimental design was a randomized complete block. On day 15, 6 piglets in each group were orally infused with 100 mL (10^9^ CFU/mL) of ETEC K88 solution, and another 6 piglets were orally infused with 100 mL of the PBS. Then, the experimental design became a 2 × 2 factorial design. The replacement ratio of FSBM was chosen based on preliminary study [35]. The experimental diets were formulated to contain equal standardized ileal digestible (SID) amino acids and digestible energy (DE) concentrations according to National Research Council-recommended nutrient requirements for 7–11 kg pigs [36] (Table 7). The SID of amino acids and DE values of SBM and FSBM were obtained from Wang et al. [37].

**Table 7:** Ingredients and nutrient composition of experimental diets (as-fed basis)^a,b^

Items	- FSBM	+ FSBM
Ingredients (%)		
Corn	60.66	60.66
Soybean meal (SBM)	24.00	18.00
Fermented soybean meal (FSBM)	-	6.00
Fish meal	4.00	4.00
Whey powder	8.00	8.00
Dicalcium phosphate	0.75	0.75
Limestone	0.80	0.80
Salt	0.35	0.35
L-Lysine HCl (78%)	0.20	0.20
Threonine	0.04	0.04
Choline chloride	0.20	0.20
Vitamin-mineral premix^b^	1.00	1.00
Total	100.00	100.00
Nutrient composition^c^		
Digestible energy (Mcal/kg)	14.31	14.25
Crude protein	19.83	20.28
Calcium	0.73	0.70
Total phosphorus	0.68	0.67
SID lysine	1.07	1.06
SID methionine	0.28	0.29
SID threonine	0.65	0.65
SID tryptophan	0.19	0.19

^a^ - ETEC K88 = infusing the phosphate buffer saline; + ETEC K88 = infusing the ETEC K88; - FSBM = control diet with 24% soybean meal; + FSBM = test diet with 6% FSBM added at the expense of soybean meal

^b^ Premix supplied per kg diet: vitamin A, 12,000 IU; vitamin D3, 2,500 IU; vitamin E, 30 IU; vitamin K3, 3 mg; vitamin B12, 0.012 mg; niacin, 40 mg; pantothenic acid, 15.0 mg; choline chloride, 400 mg; Zn, 80 mg; Mn, 30 mg; Fe, 90 mg; Cu, 10 mg; I, 0.35 mg; Se, 0.30 mg

^c^ Crude protein, calcium, and phosphorus were analyzed values, digestible energy and SID of amino acids were calculated values. The SID of amino acids and digestible energy values used for SBM and FSBM were obtained from Wang et al. (2014b), whereas those values for fishmeal and whey powder were obtained from the National Research Council (2012)

*SID* Standardized ileal digestibility

All piglets were housed individually in the metabolism cages (1.5 × 0.7 × 1.0 m^3^) equipped with a plastic feeder and nipple water drinker. The ETEC K88 challenged and unchallenged piglets were arranged in two identical rooms to avoid cross-contamination between groups. The room lighting was natural and the room temperature was controlled (25 °C ± 3 °C). Piglets were fed four times daily at 08:00, 12:00, 16:00 and 20:00, and water was available *ad libitum*.

### Growth performance

At 08:00 of day 1, 15, and 21, all piglets were weighed to determine initial body weight (BW) and final BW. Feed consumption was recorded daily during the trial. Based on these data, the ADG, ADFI and FCR were calculated.

### Fecal consistency

After the ETEC K88 challenge, fecal scores were assessed daily resulting in a total of 6 observations per piglets. The following criteria were used to score the faeces: 1, watery, mucous-like; 2, loose, semi-liquid; 3, soft, partially formed; 4, slightly soft; and 5, hard. The fecal consistency was calculated as [(Σ fecal scores for 6 days ETEC K88 challenge)/n] [38].

### Sample collection

On day 21, after an overnight fast, blood samples were obtained from each piglet by puncturing the jugular vein. The blood samples were collected into 5 mL vacutainer tubes (BD, K_2_-EDTA). After centrifuging at 3500 × ***g*** for 10 min, plasma samples were collected and stored at -80℃ for further assays. Then, all piglets were humanely euthanized with pentobarbital sodium (15 mg/kg BW). The digesta from cecum (from the terminal end) were individually collected into 10 mL sterile polypropylene centrifuge tubes, snapped frozen in liquid nitrogen, and kept at -80℃ until DNA extraction.

### Plasma cytokines analysis

The plasma cytokines (TNF-α, IL-1β, IL-6, and IL-6) concentrations were measured with ELISA kits (Wuhan ColorfulGene Biological Technology Co., Ltd., Wuhan, China), and the analysis procedures were conducted following the manufacturer’s instructions.

### Polymerase chain reactions amplicon production and high-throughput sequencing

Total bacterial DNA was extracted from the cecal digesta sample using the QIAamp DNA Stool Mini Kit (QIAGEN, Germany) according to the manufacturer’s protocols. The DNA purity was monitored on 2% agarose gels. The quantity of DNA was measured using a NanoDrop 1000 spectrophotometer. 16S rRNA genes were amplified by polymerase chain reaction (PCR) from the genomic DNA samples using specific primers for the V4 region of the bacterial 16S rRNA. The primer sequences were 515F (GTGBCAGCMGCCGCGGTAA) and 806R (GGACTACHVGGGTWTCTAAT). The PCR amplification reaction system was as follows: 1.0 µL DNA template (10 ng), 1.0 µL of each primer, 15 µL of Phusion High-Fidelity PCR Master Mix (New England Biolabs, Ipswich, USA), and 12 µL ddH_2_O in a final volume of 30 µL. The PCR thermal profile was consisted of an initial denaturation at 98 °C for 30 s, 30 cycles of denaturation at 98 °C for 10 s, annealing at 50 °C for 30 s, and extension at 72 °C for 30 s; the sample was then incubated at 72 °C for 10 min. Then, the mixture of PCR products was purified using the QIA quick Gel Extraction Kit (Qiagen, Germany) and quantified by Qubit and qPCR. Purified amplicons were pooled in equimolar and sequenced using an Illumina HiSeq 2500 platform at Novogene Bioinformatics Technology Co., Ltd. (Beijing, China).

### Bioinformatics analysis

Raw tags were de-multiplexed and quality-filtered using QIIME (V 1.9.1) with the following criteria: the 250 bp (bp) reads were truncated at any site that obtained an average quality score < 20 over a 10 bp sliding window, and the truncated reads shorter than 50 bp were discarded; exact barcode matching, two nucleotide mismatch in primer matching, and reads containing ambiguous characters were removed; only overlapping sequences longer than 10 bp were assembled according to their overlap sequence. The effective tags were obtained by comparing with the reference database using the UCHIME algorithm to truncate chimera sequences. Repetitive sequences were removed from the effective tags to acquire representative sequences using UPARSE (V 7.0.1001). The representative sequences were then ranked according to size, and those representative sequences with a size of 1 were discarded. The similarity of sequences at 97% was defined as an operational taxonomic unit (OTU). The representative OTUs sequences were annotated using RDP classifier (V 2.2) and Greengenes database (V 13.5). The data from all of the cecal digesta samples were then normalized for the analysis of alpha and beta diversity. The various alpha diversity indexes, including Observed_species, Shannon, Chao1, ACE, and Simpson index, were used. Unweighted UniFrac distance metrics were used to generate principal coordinate analysis (PCoA) plot. The relative abundance at phylum and genus levels was compared among the four groups, the top10 most abundant genera were defined as predominant genera, and sorted for the comparison.

### Statistical analysis

Data were tested for normality using the UNIVARIATE procedure of the SAS 9.2 (SAS Institute, Gary, NC). The results were showed as least squares means and standard error of the mean (SEM). Individual piglet served as the experimental unit. Data for the growth performance of piglets during the first 14 days of trial was assessed using the unpaired t test. After the ETEC K88 challenge, data were analyzed using the PROC MIXED procedure of the SAS 9.2 (SAS Institute, Gary, NC) with a 2 × 2 factorial arrangement of treatments in a randomized block design. The model included terms for the fixed effects of ETEC K88 challenge, dietary FSBM replacement and their interaction, and block was considered random effect. Differences between least squares means due to the interaction between ETEC K88 challenge and dietary FSBM replacement were tested using Duncan’s procedure. Statistical significance was defined at *p* < 0.05.

## Data Availability

The datasets used and/or analyzed during the current study are available from the corresponding author on reasonable request.

## Abbreviations

ADG: Average daily gain
ADFI: Average daily feed intake
BW: Body weight
bp: Base pair
DE: Digestible energy
ETEC K88: Enterotoxigenic *Escherichia coli* K88
ELISA: Enzyme-linked immunosorbentassay
FSBM: Fermented soybean meal
FCR: Feed conversion ratio
GIT: Gastrointestinal tract
IL-10: Interleukin-10
IL-1β: Interleukin-1β
IL-6: Interleukin 6
PBS: Phosphate-buffered saline
PCoA: Principal coordinate analysis
OUT: Operational taxonomic unit
TNF-α: Tumor necrosis factor α
SBM: Soybean meal
SID: Standardized ileal digestible
SEM: Standard error of the mean
